# Gait disorder as a predictor of spatial learning and memory impairment in aged mice

**DOI:** 10.7717/peerj.2854

**Published:** 2017-01-05

**Authors:** Xin Wang, Qing M. Wang, Zhaoxiang Meng, Zhenglu Yin, Xun Luo, Duonan Yu

**Affiliations:** 1 Department of Rehabilitation, Clinical Medical College ,Yangzhou University, Northern Jiangsu Province Hospital, Yangzhou, Jiangsu, China; 2 Stroke Biological Recovery Laboratory, Harvard Medical School, Boston, the United States of America; 3 Jiangsu Key Laboratory of Integrated Traditional Chinese and Western Medicine for Prevention and Treatment of Senile Disease, Yangzhou University, Yangzhou, China; 4 Non-coding RNA Center, Yangzhou University, Yangzhou, China; 5 Department of Rehabilitation Medicine, Nan’ao People’s Hospital of Shenzhen, Shenzhen, China; 6 Institute of Comparative Medicine, Yangzhou University, Yangzhou, China; 7 Jiangsu Co-Innovation Center for Prevention and Control of Important Animal Infectious Disease and Zoonoses, Yangzhou University, Yangzhou, China

**Keywords:** Gait disorders, Morris water maze test, Quantitative gait assessment, Cognitive impairment, Aged mice, Stride length, Coefficient of variation, Base of support, Cadence, Average speed

## Abstract

**Objective:**

To investigate whether gait dysfunction is a predictor of severe spatial learning and memory impairment in aged mice.

**Methods:**

A total of 100 12-month-old male mice that had no obvious abnormal motor ability and whose Morris water maze performances were not significantly different from those of two-month-old male mice were selected for the study. The selected aged mice were then divided into abnormal or normal gait groups according to the results from the quantitative gait assessment. Gaits of aged mice were defined as abnormal when the values of quantitative gait parameters were two standard deviations (SD) lower or higher than those of 2-month-old male mice. Gait parameters included stride length, variability of stride length, base of support, cadence, and average speed. After nine months, mice exhibiting severe spatial learning and memory impairment were separated from mice with mild or no cognitive dysfunction. The rate of severe spatial learning and memory impairment in the abnormal and normal gait groups was tested by a chi-square test and the correlation between gait dysfunction and decline in cognitive function was tested using a diagnostic test.

**Results:**

The 12-month-old aged mice were divided into a normal gait group (*n* = 75) and an abnormal gait group (*n* = 25). Nine months later, three mice in the normal gait group and two mice in the abnormal gait group had died. The remaining mice were subjected to the Morris water maze again, and 17 out of 23 mice in the abnormal gait group had developed severe spatial learning and memory impairment, including six with stride length deficits, 15 with coefficient of variation (CV) in stride length, two with base of support (BOS) deficits, five with cadence dysfunction, and six with average speed deficits. In contrast, only 15 out of 72 mice in the normal gait group developed severe spatial learning and memory impairment. The rate of severe spatial learning and memory impairment was significantly higher in the abnormal gait group as compared to that in the normal gait group (*x* = 21.986, *P* < 0.001). All five parameters used to assess gait predicted severe spatial learning and memory impairment in aged mice (*P* < 0.01). However, the difference of the area under the ROC (receiver operating characteristic) curve for each quantitative gait parameter was not statistically significant.

**Conclusion:**

Gait disorders are a predictor of severe spatial learning and memory impairment in aged mice, and stride length, variability of stride length, base of support, cadence, and average speed are all sensitive parameters for assessing gait.

## Introduction

Walking is considered an automatic process that involves little or no cognitive input, especially in young people. However, there is evidence that walking may involve cognitive functions, such as memory, attention, and executive functions (Akl & Mihailidis, 2015; Wrightson, Ross & Smeeton, 2016). Some seniors walk slowly or fall even though the function of their motion systems seems normal. Recent studies show that some seniors with poor walking performance had cognitive impairment (Hashimoto et al., 2014). However, there are few studies to explore the relationship of abnormal gait and cognitive impairment in the elderly.

As most gait disorders are mild, it is difficult for clinicians to detect them in advance. Thus, it is necessary to build a standardized or validated gait assessment protocol. Gait is considered a complex motor behavior with many measurable parameters including stride length and average speed (Djuric-Jovicic et al., 2014; Verghese et al., 2007). However, it is currently unknown which gait parameters can predict the development of cognitive impairment.

In this study, we utilized quantitative gait assessment to investigate the difference in walking gait between aged and young mice. Our aim was to investigate whether quantitative gait assessment could evaluate the risk of severe spatial learning and memory impairment in aged mice with no substantial spatial learning and memory impairment at baseline. We also investigated the relationship between quantitative gait parameters and decline in cognitive function. Taken together, these tests may provide a new and simple way to identify patients who are at a high risk for cognitive impairment and dementia, and could facilitate the development of novel preventive strategies for seniors with cognitive impairment.

## Materials and Methods

### Mice

All animal experiments were approved by the Animal Care and Use Committee of the School of Medicine in Yangzhou University (License 2013-10-03). Male Kunming mice (Two and 12 months old) were obtained from the Experimental Animal Center of Yangzhou University and were maintained at a temperature of 25 °C with a 12-hour light-dark cycle and free access to food and water.

### Experimental design

A total of 100 12-month-old male mice that had no obvious abnormal motor ability, such as inability to walk, tendency to walk in circles and abnormally slow swim speed, and whose Morris water maze performances were no significantly different from those of two-month-old male mice, were selected for study. Quantitative gait assessment of these selected aged mice was then implemented. The gaits of aged mice were defined as abnormal when the values of quantitative gait parameters were two standard deviations (SD) lower or higher than those of two-month-old male mice (Li et al., 2015). Gait parameters included stride length, variability of stride length, base of support, cadence, and average speed. The 12-month-old mice were divided into abnormal or normal gait groups according to the results of the quantitative gait assessment. After nine months in standard housing, the aged mice underwent the Morris water maze test again, and the results were compared to a new group of two-month-old mice. Mice exhibiting severe spatial learning and memory impairment were separated from the mice with no or mild cognitive dysfunction. The rate of severe spatial learning and memory impairment in the abnormal and normal gait group**s** was tested by a chi-square test and the correlation between gait dysfunction and decline in cognitive function was tested using a diagnostic test. To eliminate bias, behavior study was subjected to blind testing.

### Morris water maze test

The study involved the Morris water maze test. The apparatus and procedures of the test have been described in our previously published work (Wang et al., 2013). In short, the Morris water maze test includes the place navigation test, in which the escape latency and number of passes over a platform were used to evaluate learning and memory functions. The water temperature was maintained at a constant 19 °C with a clear plexiglass platform 2 cm below water level. In the first three days, mice were trained in the Morris water maze with a platform. The escape latency of when mice found the platform was recorded on the fourth day. On the fifth day, the platform was removed, and number of passes over platform in 60 s was recorded. Software for behavior analysis (RD; Mobiledatum, Shanghai, China) was used in the Morris water maze test.

### Quantitative gait assessment

**Figure 1:**
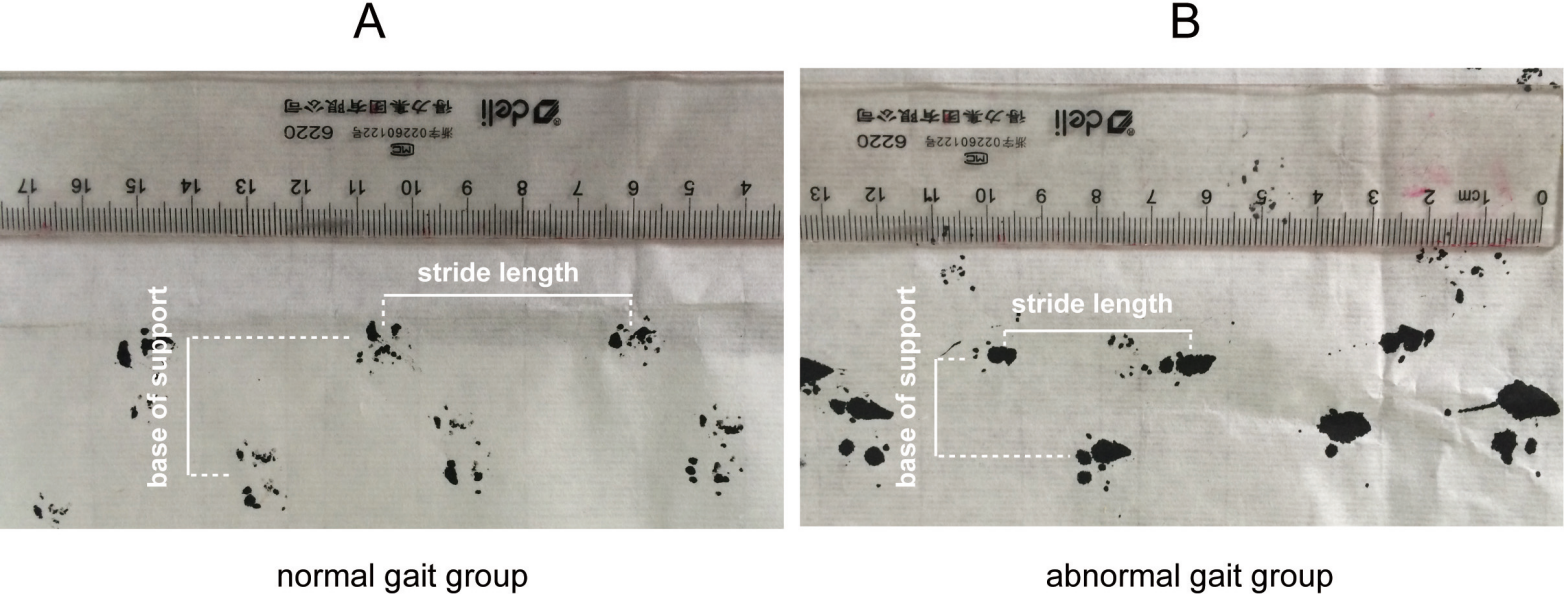
Representative stride length and base of support measurement using forelimb paw prints in the mice of normal gait group and abnormal gait group. (A) shows details of the normal stride length and base of support in normal gait group (B) shows the reduced stride length and base of support in the abnormal gait group.

Quantitative gait assessment was performed using an improved method adapted from D’Hooge et al. (Fernagut et al., 2002). The forelimb paw prints of mice were covered with a commercially available black ink pencil and mice were placed on a strip of paper (4.5 cm wide, 80 cm long) on a brightly lit runway to walk towards a dark goal box. Paw prints made at the beginning (10 cm) and end (10 cm) of the run were excluded because of velocity changes. Runs in which the mice made stops or obvious decelerations, as observed by the experimenter, were excluded from analysis. Gait parameters, including stride length, variability of stride length, base of support, cadence, and average speed were measured (Fig. 1). The table (Table 1) shows the definitions of gait parameters.

**Table 1:** Definitions of gait parameters.

Parameter	Definition
Stride length(cm)	The distance between two consecutive travels in the same paw
Variability of stride length	The coefficient of variation of stride length of every mice (standard deviation/mean) × 100%
Base of support (cm)	Distance between forelimbs at maximum area
Cadence (steps/s)	Steps per second in a trial
Average speed (cm/s)	Distance covered on two trials by the ambulation time

### Diagnosis of severe spatial learning and memory impairment

After nine months in standard housing, mice were subjected to the Morris water maze test for a second time. The control group consisted of two-month-old mice (*n* = 20). Aged mice were considered to have developed severe spatial learning and memory impairment if the values of escape latency and the number of passes over platform were 3 SD lower or higher than that of mice in the control group (Abaza et al., 2012; Li et al., 2015; Rowe et al., 1998).

### Statistical analysis

Quantitative gait parameters and the results of the Morris water maze tests were expressed as mean ± SD, and the differences between the two groups were evaluated using analysis of variance (ANOVA) followed by the Newman–Keuls multiple-range test. The classified data were analyzed using a chi-square test. The authenticity between gait parameters was examined by a diagnostic test and the area under the ROC curve of each parameter was calculated. All data were analyzed using SPSS 18.0 statistics software (SPSS, Chicago, IL, USA). The differences were considered significant if *P* < 0.05.

## Results

### Characteristic information of mice

Nine months later, five mice in the original 12-month-old mouse group died and ninety-five mice remained. The weight of all mice is listed in Table 2.

**Table 2:** Mouse weight.

Mouse group	Weight (g)
2-month-old (*n* = 20)	28.05 ± 0.69
12-month-old (*n* = 100)	39.11 ± 1.41^a^
new 2-month-old (*n* = 20)	27.70 ± 0.86
21-month-old (*n* = 95)	39.13 ± 1.42^b^

**Notes.**

a *P*-value < 0.05 (*t*-test, vs 2-month-old mice).

b *P*-value < 0.05 (*t*-test, vs new 2-month-old mice).

As shown in Table 2, the weight of 12-month-old mice and 21-month-old mice were remarkably higher than those of 2-month-old mice (*P* < 0.05), while the weight of 21-month-old mice were no significantly different than those of 12-month-old mice (*P* < 0.05).

### Quantitative gait parameters at baseline

Of the 100 aged mice, 25 exhibited gait disorders at baseline, including 10 with stride length deficits, 19 with CV of stride length, eight with BOS deficits, 12 with cadence dysfunction, and 13 with average speed deficits (Fig. 1). Any mouse may exhibit multiple abnormal parameters. Accordingly, aged mice were divided into a normal gait group (*n* = 75) and an abnormal gait group (*n* = 25). As shown in Fig. 1 and Table 3, stride length, CV of stride length, BOS, cadence, and average speed were significantly different between the normal gait and abnormal gait groups (*P* < 0.05).

**Table 3:** Quantitative gait parameters at baseline.

	Control group young (*n*)	Aged mice	
		Normal(*n*)	Abnormal(*n*)
Stride length (cm)	5.28 ± 0.47 (*n* = 20)	4.84 ± 0.35 (*n* = 90)	3.88 ± 0.50 (*n* = 10)^a^
CV of stride length (%)	3.43 ± 0.33 (*n* = 20)	3.53 ± 0.28 (*n* = 81)	5.68 ± 0.22 (*n* = 19)^a^
Base of support (cm)	2.78 ± 0.19 (*n* = 20)	2.68 ± 0.17 (*n* = 92)	2.15 ± 0.14 (*n* = 8)^a^
Cadence (steps/s)	9.58 ± 0.58 (*n* = 20)	9.62 ± 0.58 (*n* = 88)	7.57 ± 0.54 (*n* = 12)^a^
Average speed (cm/s)	44.35 ± 399 (*n* = 20)	44.82 ± 4.05 (*n* = 87)	29.08 ± 4.06 (*n* = 13)^a^

**Notes.**

a *P*-value < 0.05 (*t*-test, vs control group).

### The development of severe spatial learning and memory impairment in normal and abnormal gait groups

After nine months, three mice in the normal gait group and two mice in the abnormal gait group had died. The Morris water maze test revealed that 17 out of 23 mice in the abnormal gait group had developed severe spatial learning and memory impairment, including six with deficits in stride length, 15 with CV of stride length, two with BOS dysfunction, five with cadence dysfunction, and six with average speed deficits. In contrast, only 15 out of 72 mice in the normal gait group had developed severe spatial learning and memory impairment (Table 4). The difference between the two groups was statistically significant (*x* = 21.986, *P* < 0.001).

**Table 4:** Rate of severe spatial learning and memory impairment in normal and abnormal gait groups.

	Normal gait	Abnormal gait
Impaired cognition	15	17
Normal cognition	57	6

**Figure 2:**
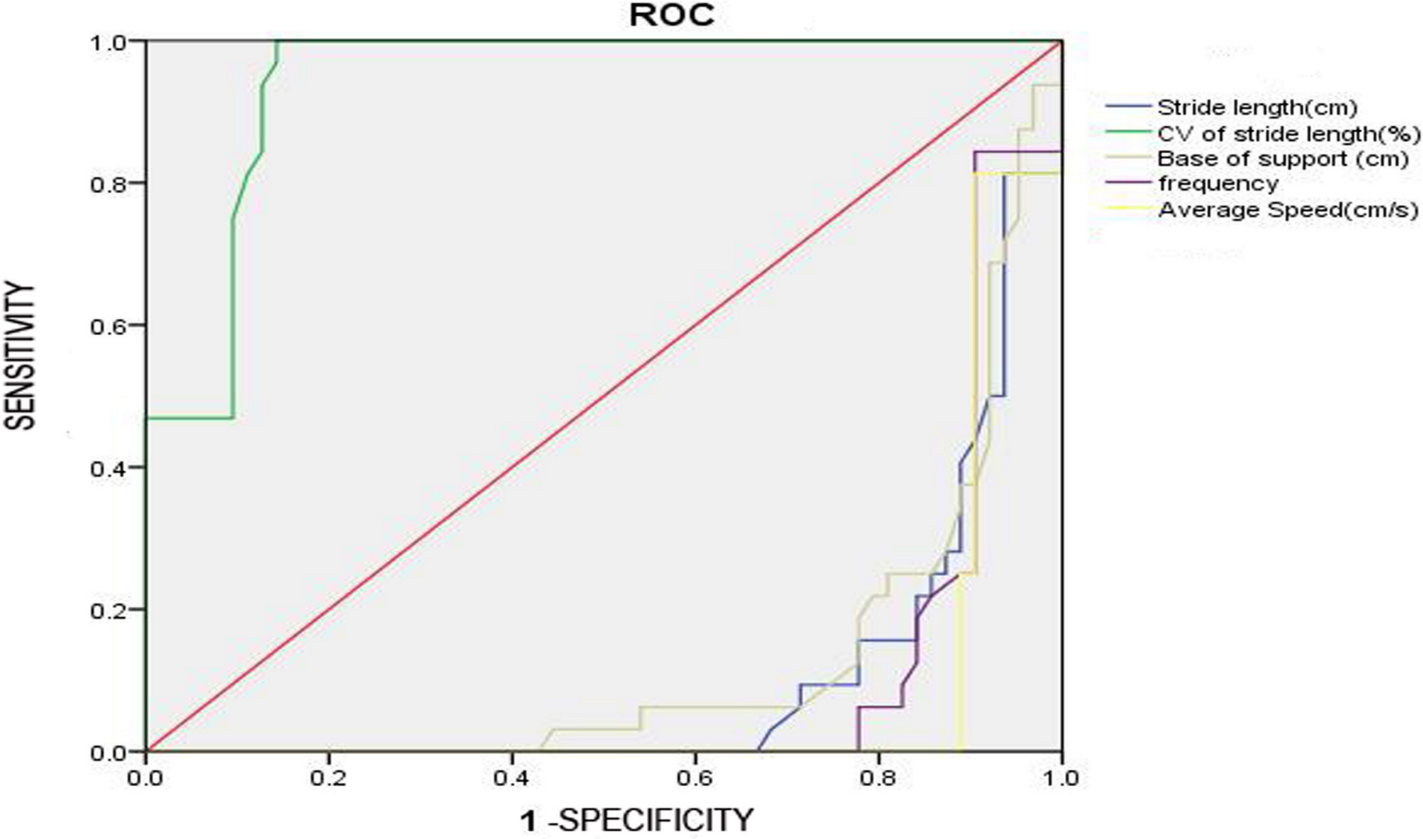
The ROC curve. The figure shows that area under the ROC curve of each of the five quantitative gait parameters is not statistically significant (P > 0.05).

To examine the relationship between quantitative gait parameters and cognitive function, the area under the ROC curve of each of the five quantitative gait parameters was calculated. As shown in Table 5 and Fig. 2, all five parameters could predict severe spatial learning and memory impairment in aged mice (*P* < 0.01), while the area under the ROC curve of each of the five quantitative gait parameters is not statistically significant (*P* > 0.05), indicating that the five quantitative gait parameters used to predict severe spatial learning and memory impairment are not statistically different.

**Table 5:** Relationship between quantitative gait parameters and cognitive function.

Parameter	*P* value	Area under the ROC curve	95% CI
Stride length (cm)	<0.001	.897	0.835, 0.959
CV of stride length	<0.001	.942	0.898, 0.986
Base of support (cm)	<0.001	.874	0.802, 0.945
Cadence (steps/s)	<0.001	.900	0.836, 0.965
Average speed (cm/s)	<0.001	.919	0.858, 0.980

### The results of Morris water maze test

As shown in Table 6, the Morris water maze performances of 100 12-month-old male mice were no significantly different than those of two-month-old male mice. After nine months, the Morris water maze performances of the selected 100 12-month-old male mice had changed. The values of escape latency and the number of passes over the platform of 32 21-month-old mice with severe spatial learning and memory impairment were 3 SD lower or higher than that of new 2-month-old mice, while the values of escape latency and the number of passes over the platform of 63 21-month-old mice without severe spatial learning and memory impairment were not 3 SD lower or higher than that of new 2-month-old mice. There was no significant difference in swimming speed between young mice and aged mice (*P* > 0.05).

**Table 6:** Results of Morris water maze test.

	Escape latency (sec)	Numbers of pass over platform	Swimming speed (cm/sec)
2-month-old mice (*n* = 20)	39.48 ± 6.91	4.50 ± 1.57	17.97 ± 2.15
12-month-old mice (*n* = 100)	39.76 ± 2.11	4.48 ± 0.50	16.68 ± 1.44
new 2-month-old mice (*n* = 20)	40.77 ± 6.15	4.30 ± 1.34	17.98 ± 2.69
21-month-old mice without severe spatial learning and memory impairment (*n* = 63)	48.27 ± 4.96	2.70 ± 0.75	15.81 ± 1.38
21-month-old mice with severe spatial learning and memory impairment (*n* = 32)	59.95 ± 0.12	0.09 ± 0.29	15.47 ± 1.15

## Discussion

The relationship between gait and cognition in the elderly population has drawn increased attention in recent years. Studies on early stage Parkinson’s disease have found that a patient’s cognitive functions, such as attention and memory, can affect the patient’s gait (Lord et al., 2014). Other studies have demonstrated that abnormal gait could be a predictor of non-Alzheimer’s dementia (Verghese et al., 2002). Thus, complex high-level cerebral functions can affect gait of walking. In clinical research, it is difficult to identify how a decline in cognitive function leads to abnormal gait as baseline cognitive functioning varies between patients (Verghese et al., 2007). It is also difficult to identify which gait parameters can be used to predict a cognitive decline. In this study, we selected a group of young and aged mice that exhibited similar baseline scores on the Morris water maze. Because some of the aged mice with substantial spatial learning and memory impairment as well as abnormal motor ability had more obvious gait disorder, these mice were eliminated in order to explore the direct relationship between gait and cognition. Based on gait assessment results, aged mice were divided into abnormal gait and normal gait groups. Nine months later, the rate of severe spatial learning and memory impairment in the abnormal gait group of aged mice was significantly higher than that of those in the normal gait group. Our findings supported the viewpoint that the presence of gait abnormalities strongly predicts cognitive impairment (Kikkert et al., 2016).

Walking is a cognitive process which requires higher-level control and is not an automatic movement (LeMonda et al., 2015). Studies have demonstrated that executive functions, attention and memory are closely related to walking (Dalton, Sciadas & Nantel, 2015). Executive functions refer to a variety of higher cognitive processes that modulate and use information from the posterior cortical sensory systems to produce behavior (Honan et al., 2015). These include initiation or intention of action, planning, attention, and so on. Attention is a dynamic function driven by sensory perception and the need to select a preferred stimulus for a particular action while ignoring the unnecessary and irrelevant (Olk, Peschke & Hilgetag, 2015). Memory represents the brain’s ability to recognize, maintain, and recall acquired information. The execution of any acquired action, including walking, requires continued sensory input from the posterior cortical sensory systems, which is integrated with previously acquired motor function to constantly adjust acquired actions (Derdikman et al., 2006; Vingerhoets, 2014; Yuan & Hou, 2015). Useful information for a specific action is utilized, while useless information is abandoned. Thus, many cognitive functions could influence the gait of walking of humans or animals, including aged mice. The Morris water maze mainly assesses hippocampus-dependent spatial learning and memory but not complete cognitive function. Walking requires the efficient integration of many neural systems, including motor, sensory, and cognitive processes, and cognitive sub-systems such as memory, attention, and executive function (Akl & Mihailidis, 2015; Allali et al., 2012). Thus, mice that scored the same in the Morris water maze may have different gait scores. Despite similar scores in the Morris water maze at baseline, the rates of severe spatial learning and memory impairment in the abnormal gait group after nine months were significantly higher than that of those in the normal gait group, suggesting that quantitative gait assessment is more sensitive than the Morris water maze in predicting severe spatial learning and memory impairment in aged mice. In aged mice, lacunar infarction, leukoaraiosis, inflammatory cytokines, and neurodegenerative disorders can damage brain function. In the early stage, these factors lead to little damage of cortical-subcortical neural circuits and only mild executive function decline, which can not be detected by the Morris water maze. But this cognitive decline can be detected by quantitative gait assessment and abnormal gait became a prominent symptom. (Morein-Zamir et al., 2016; Rosano et al., 2007; Suchy, Lee & Marchand, 2013) As time goes by, the damage of cortical-subcortical neural circuits of the aged mice may spread further to affect cognitive areas such as the hippocampus and throughout the neural circuits, leading to a more severe decline in function. At this time, the cognitive decline can be detected by the Morris water maze (Ball et al., 2010).

When gait impairments are subtle, they may not be detected by the untrained eye, so quantitative measures provide an objective means of assessing gait that minimizes examiner bias. Based on footfalls recorded on the walkway, we obtained quantitative gait parameters, including the stride length, CV of stride length, base of support, cadence and average speed. Studies show that increased levels of attention and memory are associated with quicker average speed and cadence, while the stride length, CV of stride length, and base of support are thought to be related to executive functions, which chiefly correspond with the coefficient of variance (Akl & Mihailidis, 2015; Verghese et al., 2009). Studies indicate that as people get older, their executive function declines, and both their mean stride length and base of support also decrease (Bridenbaugh & Kressig, 2014). At the same time, as their motor control decreases, the variation of their stride length increases (Jahn, Zwergal & Schniepp, 2010). Our study indicates that if the aged mice with relatively normal motor ability had no significant difference in spatial learning and memory, abnormal gait may be associated with the decline of executive function and attention. Our study also suggests that all five parameters of quantitative gait assessment could be used to predict severe spatial learning and memory impairment in aged mice.

In order to understand which parameter is the most accurate in predicting severe spatial learning and memory impairment, we conducted a correlation analysis between the five parameters and cognitive impairment. Results show that the five parameters exhibit the same sensitivity in predicting severe spatial learning and memory impairment. The possible reason is that these parameters are measured without changes in spatial memory, and moreover, the results primarily reveal the influence of attention and executive function on gait (Bridenbaugh & Kressig, 2014; Jahn, Zwergal & Schniepp, 2010). As the parameters all reflect the same function, it is expected that the difference among these parameters are very small and not significant.

This study is also important for clinical practice. First, it has been shown that normal gait needs not only good motor ability but also good cognitive ability. Second, the abnormal data from quantitative gait test can be used as a predictive parameter of high-risk falls. Third, the incidence of cognitive impairment in clinical practice can be predicted by measuring stride length, cadence, and average speed of elderly individuals who do not exhibit memory impairment at baseline. Lastly, assessing one parameter may be enough to predict cognitive impairment, which greatly improves its clinical feasibility.

However, it remains unknown whether quantitative gait assessment parameters can be used to predict cognitive impairment when memory is different at baseline; further studies are needed for answering such questions.

## Conclusions

Gait disorders are a predictor of severe spatial learning and memory impairment in aged mice, and stride length, variability of stride length, base of support, cadence, and average speed are all sensitive parameters for assessing gait.

##  Supplemental Information

10.7717/peerj.2854/supp-1 Supplemental Information 1 Raw data: average speed of quantitative gait assessment This data was used for data analyses and preparation for Figs. 1 and 2 and Tables 3–5.

10.7717/peerj.2854/supp-2 Supplemental Information 2 Raw data: base of support of quantitative gait assessment This data was used for data analyses and preparation for Figs. 1 and 2 and Tables 3–5.

10.7717/peerj.2854/supp-3 Supplemental Information 3 Raw data: cadence of quantitative gait assessment This data was used for data analyses and preparation for Figs. 1 and 2 and Tables 3–5.

10.7717/peerj.2854/supp-4 Supplemental Information 4 Raw data: CV of stride length of quantitative gait assessment This data was used for data analyses and preparation for Figs. 1 and 2 and Tables 3–5.

10.7717/peerj.2854/supp-5 Supplemental Information 5 Raw data: stride length of quantitative gait assessment This data was used for data analyses and preparation for Figs. 1 and 2 and Tables 3–5.

10.7717/peerj.2854/supp-6 Supplemental Information 6 Raw data: first water maze test of mice This data was used for data analyses and preparation for Figs. 1 and 2 and Tables 3–5.

10.7717/peerj.2854/supp-7 Supplemental Information 7 Raw data: second water maze test of mice This data was used for data analyses and preparation for Figs. 1 and 2 and Tables 3–5.

10.7717/peerj.2854/supp-8 Supplemental Information 8 Abnormal gait 1 The result of abnormal gait. This data was used for data analyses and preparation for Fig. 1 and Table 3.

10.7717/peerj.2854/supp-9 Supplemental Information 9 Abnormal gait 2 The result of abnormal gait. This data was used for data analyses and preparation for Fig. 1 and Table 3.

10.7717/peerj.2854/supp-10 Supplemental Information 10 Abnormal gait 3 The result of abnormal gait. This data was used for data analyses and preparation for Fig. 1 and Table 3.

10.7717/peerj.2854/supp-11 Supplemental Information 11 Normal gait 1 The result of normal gait. This data was used for data analyses and preparation for Fig. 1 and Table 3.

10.7717/peerj.2854/supp-12 Supplemental Information 12 Normal gait 2 The result of normal gait. This data was used for data analyses and preparation for Fig. 1 and Table 3.

10.7717/peerj.2854/supp-13 Supplemental Information 13 Normal gait 3 The result of normal gait. This data was used for data analyses and preparation for Fig. 1 and Table 3.

10.7717/peerj.2854/supp-14 Supplemental Information 14 Water maze equipment

## Additional Information and Declarations

### Data Availability

The following information was supplied regarding data availability: The raw data is included in Tables 2–6.
