# Supplementary figures and images for: Gait disorder as a predictor of spatial learning and memory impairment in aged mice
Source: PeerJ. 2017 Jan 5;5:e2854. doi: 10.7717/peerj.2854 (PMC5289446; doi:10.7717/peerj.2854)

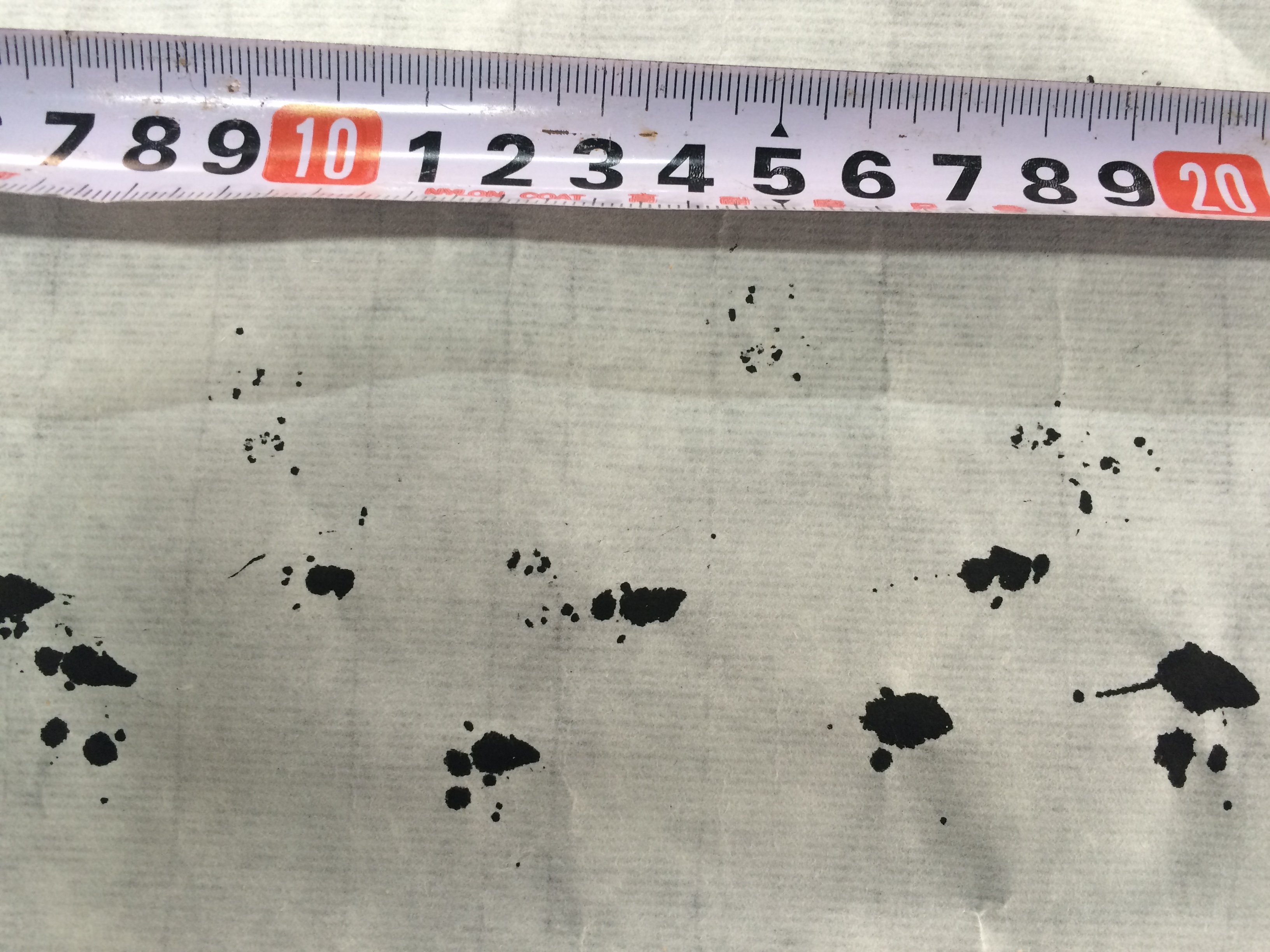

Supplement: Supplemental Information 8 — The result of abnormal gait. This data was used for data analyses and preparation for Fig. 1 and Table 3. [file peerj-05-2854-s008.jpg]

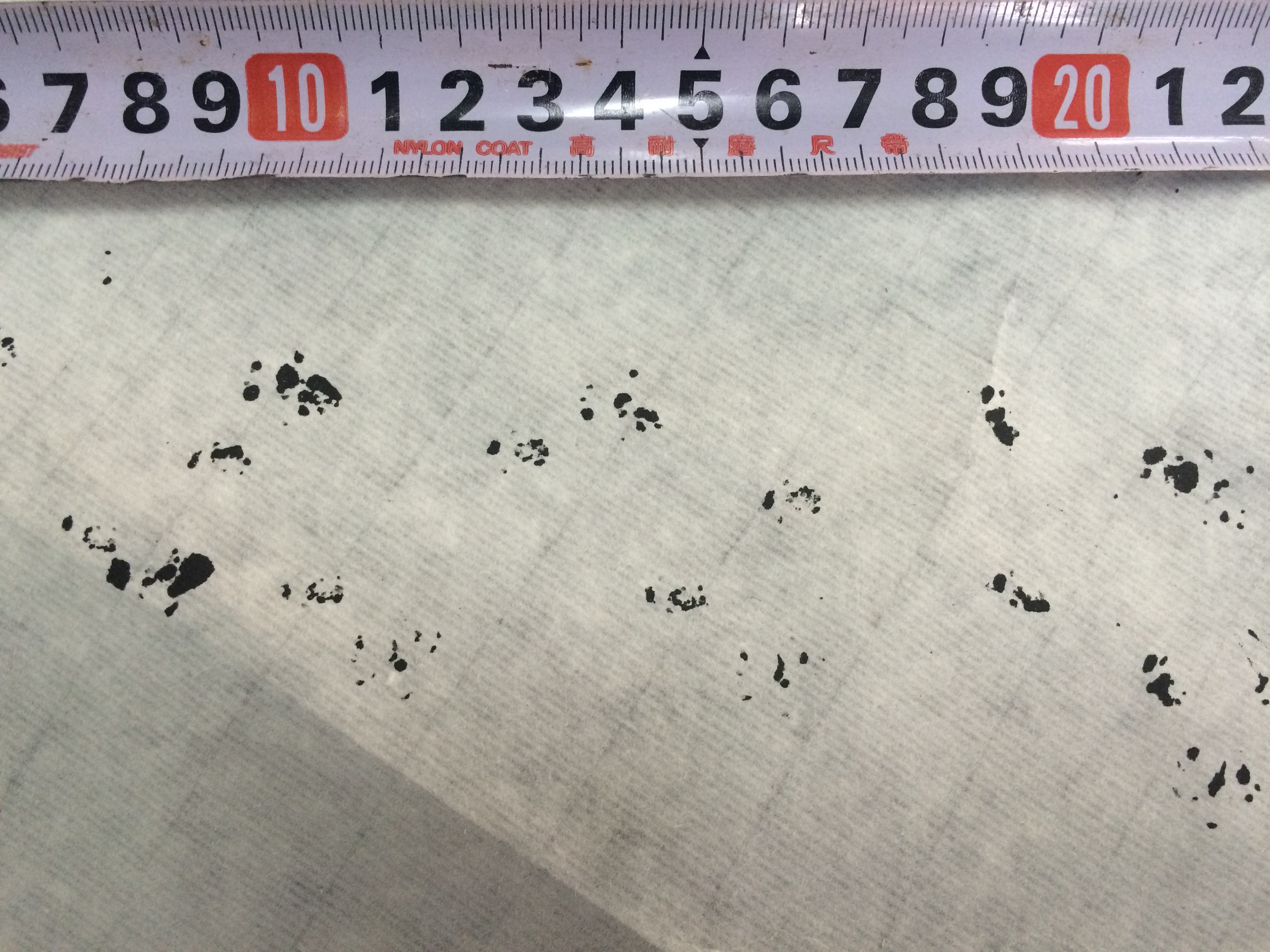

Supplement: Supplemental Information 9 — The result of abnormal gait. This data was used for data analyses and preparation for Fig. 1 and Table 3. [file peerj-05-2854-s009.jpg]

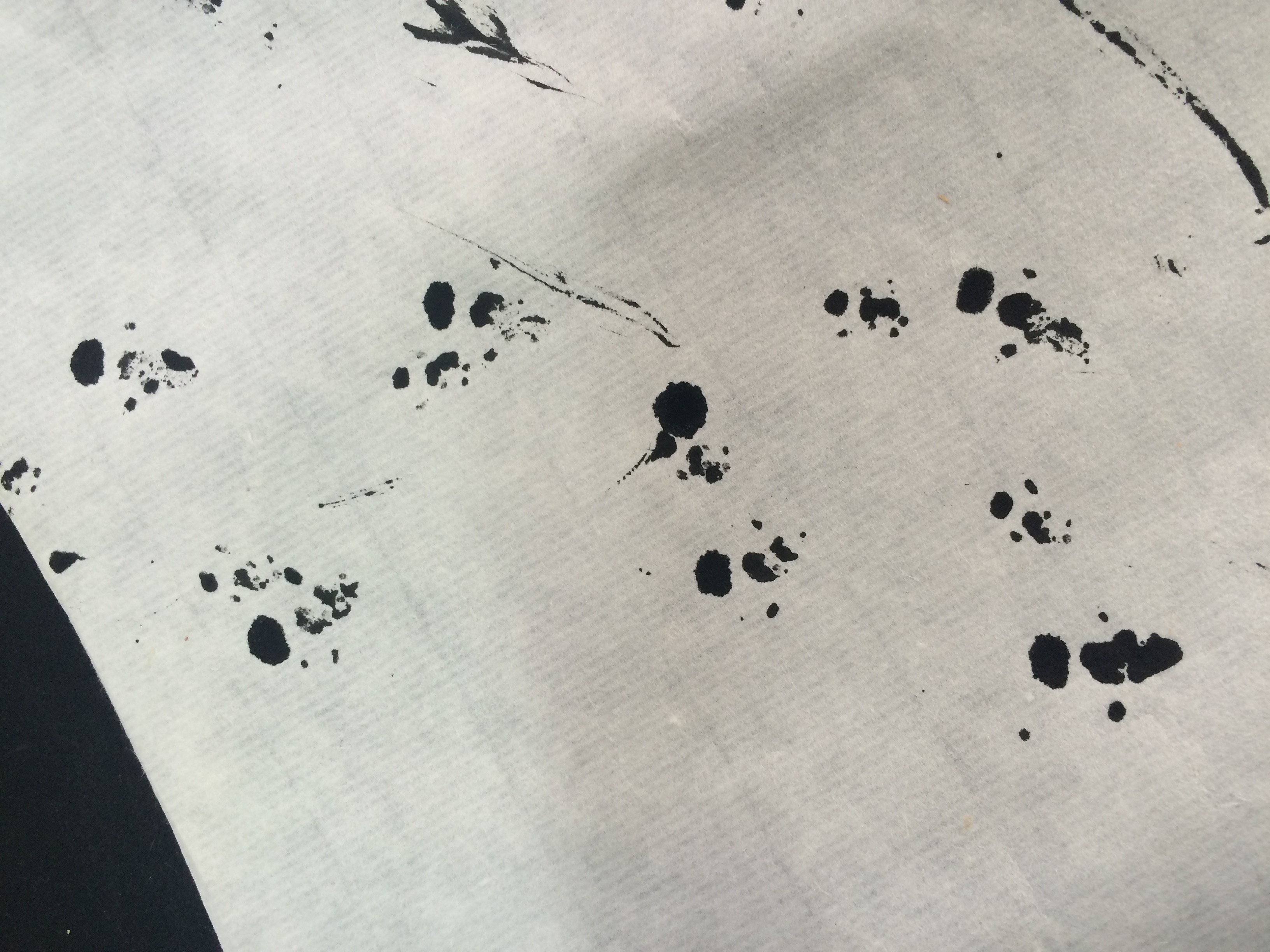

Supplement: Supplemental Information 10 — The result of abnormal gait. This data was used for data analyses and preparation for Fig. 1 and Table 3. [file peerj-05-2854-s010.jpg]

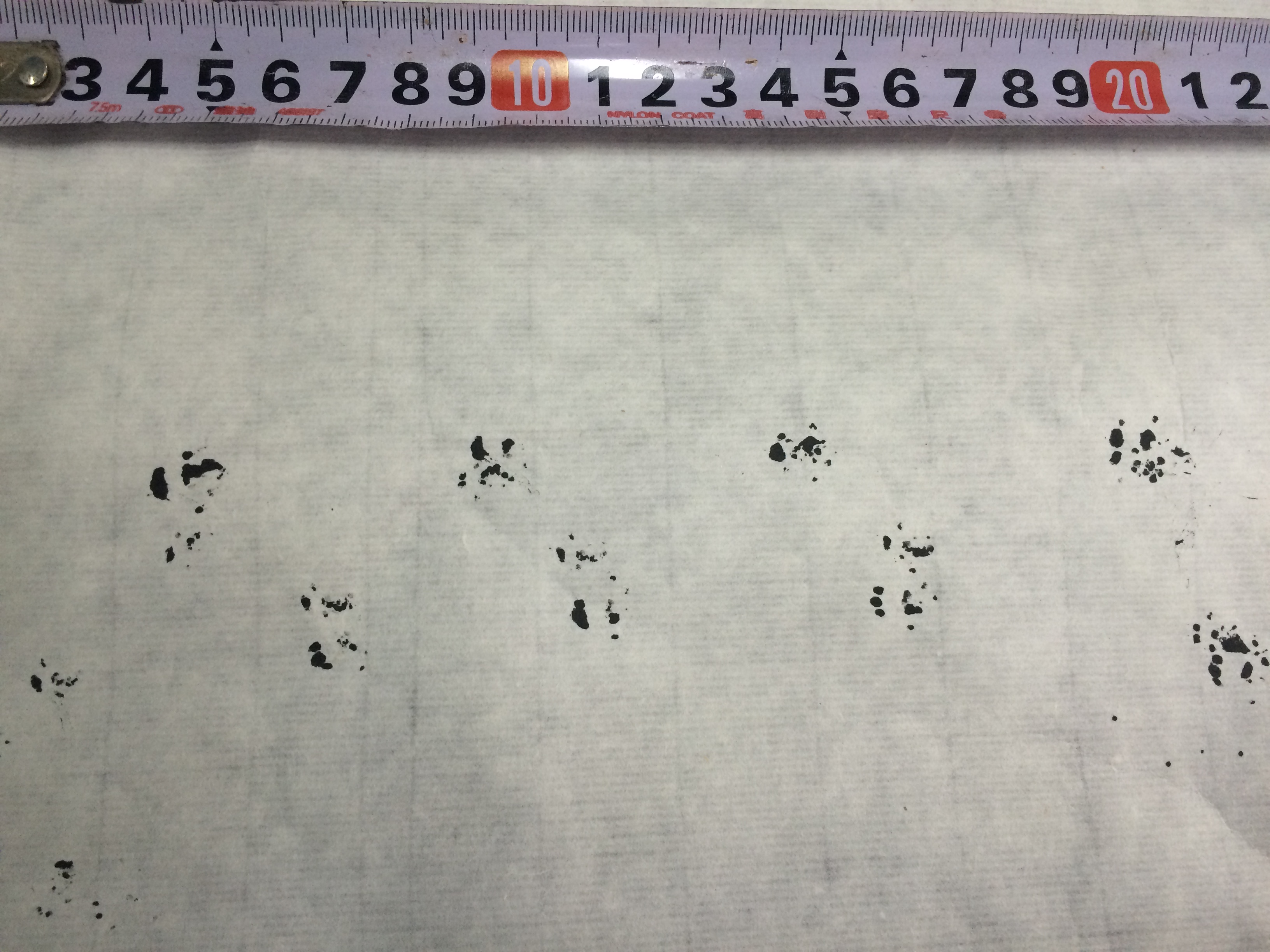

Supplement: Supplemental Information 11 — The result of normal gait. This data was used for data analyses and preparation for Fig. 1 and Table 3. [file peerj-05-2854-s011.jpg]

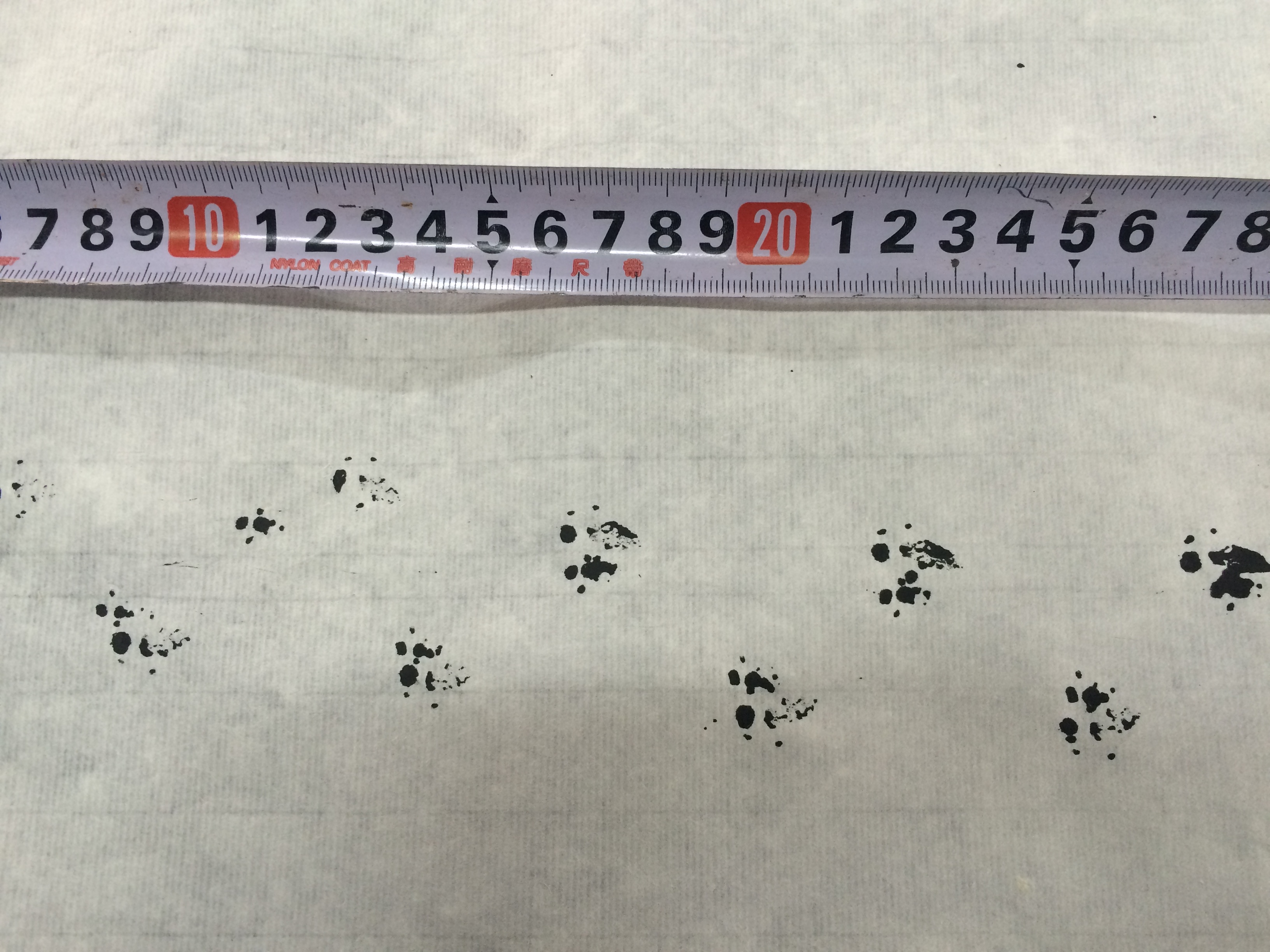

Supplement: Supplemental Information 12 — The result of normal gait. This data was used for data analyses and preparation for Fig. 1 and Table 3. [file peerj-05-2854-s012.jpg]

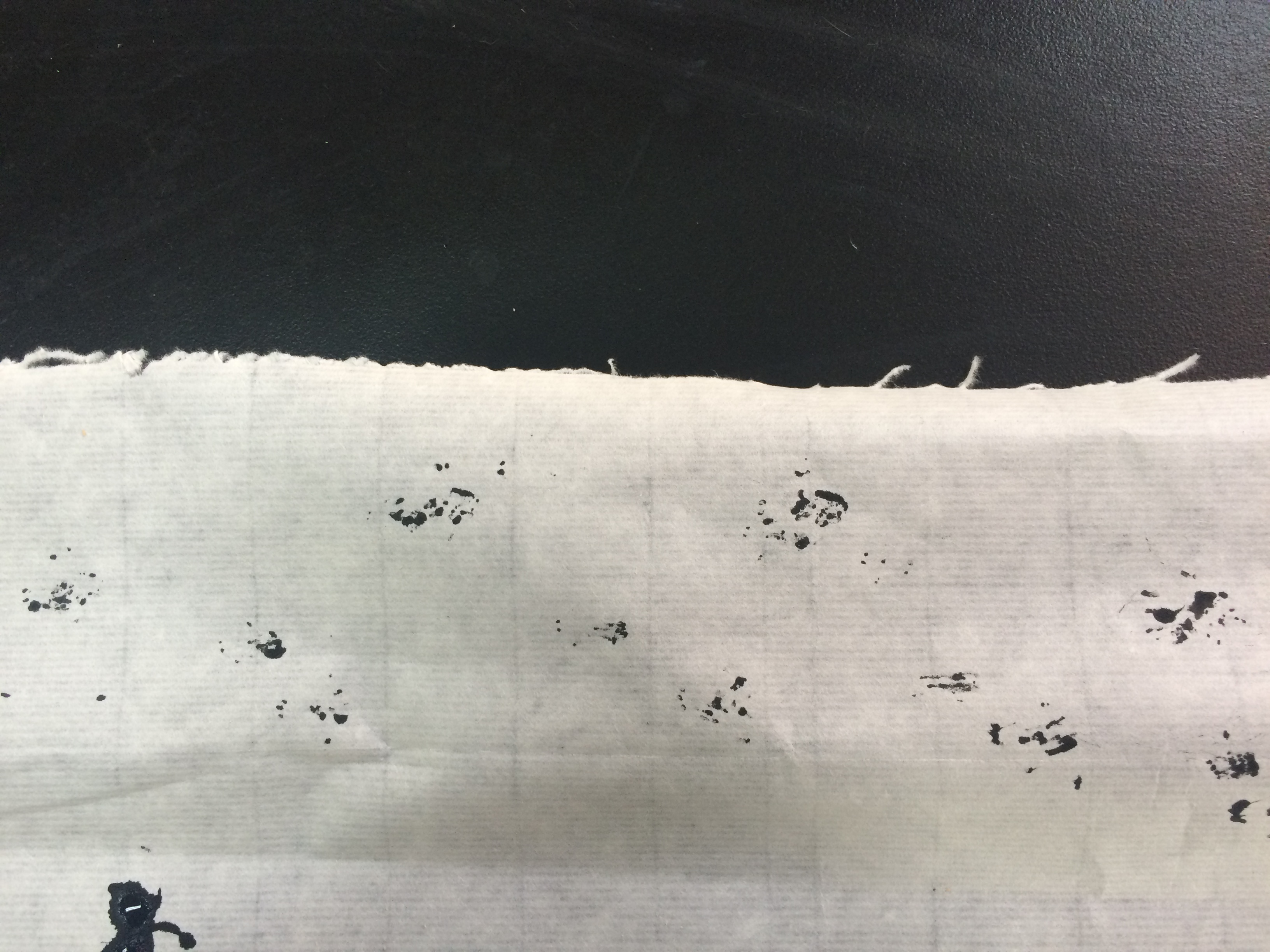

Supplement: Supplemental Information 13 — The result of normal gait. This data was used for data analyses and preparation for Fig. 1 and Table 3. [file peerj-05-2854-s013.jpg]

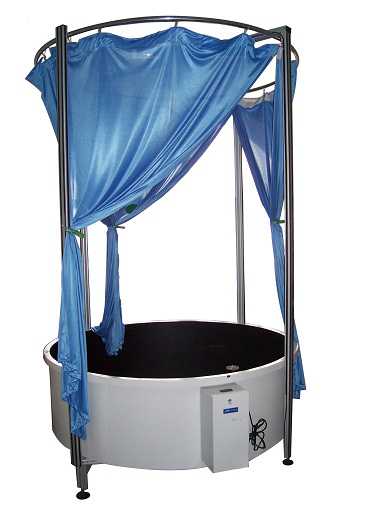

Supplement: Supplemental Information 14 [file peerj-05-2854-s014.jpg]
